# Inhibited Expression of NLRP12 Promotes the Development of Triple-Negative Breast Cancer by Activating the NF-κB Pathway

**DOI:** 10.1007/s12013-023-01166-9

**Published:** 2023-09-02

**Authors:** Wenbin Kuang, Qingdan Gu, Ying Zhou, Xiaoqin Xiao, Dabao He, Qiuchan Deng

**Affiliations:** 1 Department of Clinical Laboratory, Shenzhen Longhua District Central Hospital, Guangdong Medical University, Shenzhen, 518110 China; 2 Laboratory Medicine, Guangdong Medical University, Zhanjiang, 524023 China; 3 Department of Pathology, Shenzhen Longhua District Central Hospital, Guangdong Medical University, Shenzhen, 518110 China

**Keywords:** NLRP12, Triple-negative breast cancer, NF-κB, Proliferation, Migration, Invasion

## Abstract

NLRP12 can affect the progression of different diseases, including hepatocellular carcinoma. However, no report on triple-negative breast cancer (TNBC) has been found. Thus, this study aimed to explore the role of NLRP12 in TNBC. In our study, immunohistochemistry, real-time quantitative PCR (qPCR), and Western blot assays were used to evaluate NLRP12 expression in TNBC tissues and cells. Then, NLRP12 lentivirus was constructed and infected into MDA-MB-231 and MDA-MB-157 cells with or without PTD-p65-P1 treatment. Next, cells were collected for cell function detection using the following procedures: colony formation assay for proliferation, Transwell for migration and invasion, and Western blot for NF-κB and MAPK pathway-associated proteins. Finally, a xenograft mouse model was applied; the tumor volume and weight were determined, and NLRP12, p-IκBb-α, and p-IκBb-α expressions were evaluated using qPCR and Western blot. Results indicated that NLRP12 was lowly expressed in TNBC tissues and cells. The inhibition of NLRP12 could induce the proliferation, migration, and invasion of TNBC cells, which also could be reversed by inhibiting the NF-κB pathway (PTD-p65-P1). Moreover, silencing of NLRP12 could upregulate p-IκBb-α, while IκBb-α, p-ERK, ERK, p-p38, p38, p-JNK, and JNK expressions remained unchanged, thereby indicating that only the NF-κB pathway could be activated by NLRP12 silencing. Furthermore, the xenograft mouse model confirmed the abovementioned findings. Therefore, the low expression of NLRP12 promoted the proliferation, migration, and invasion in TNBC cells by activating the NF-κB pathway. This study might provide insights into TNBC therapy.

## Introduction

Breast cancer is a common malignant cancer with a highly heterogeneous disease in women worldwide [2]. Triple-negative breast cancer (TNBC) is a subtype of breast cancer, which is defined as the lack of progesterone receptor, estrogen receptor (ER), and human epidermal growth factor receptor 2 (HER2), and about 10–20% of breast cancer is TNBC [3]. TNBC is characterized by local infiltration, strong aggressiveness, early lymph node metastasis, easy recurrence, and high mortality, which makes clinical management more difficult [4]. It is also common in pre-menopausal women, and patients who are diagnosed with late clinical TNM stage [5]. For ER + /HER+ breast cancer, endocrine therapy and HER2-targeted therapy are the common treatment [6]. However, patients with TNBC cannot be treated using these two treatment methods and they still rely on surgery and chemotherapy, which results in poor prognosis, high metastasis rates, and mortality. Therefore, finding new therapeutic targets for TNBC is necessary.

The NLR family is a major pattern recognition receptor family of the intrinsic immune system [7, 8]. It recognizes not only pathogen-associated molecular patterns, but also damage-associated molecular patterns (DAMPs) such as heat shock proteins, purine metabolites, and reactive oxygen species [9, 10]. The NLR family pyrin domain containing 12 (NLRP12) belongs to the NLR family, consisting of the N-terminal pyrin domain (PYD), intermediate nucleotide-binding domain (NBD), NACHT-related structural domain, and C-terminal leucine-rich repeat [11]. NLRP12 can be found not only in myeloid cells, particularly in neutrophils and eosinophils, but also in various organs such as the brain, lung, spleen, liver, and small intestine [12]. Similar to NLRP3, NLRP12 has also been reported to recruit and bind downstream ASC and caspase-1, as well as form inflammasomes, thereby participating in inflammation [13]. In addition, NLRP12 can recognize multiple pathogens and sense multiple DAMPs to achieve immune defense [14]. Moreover, the specific function of NLRP12 is anti-inflammatory. NLRP12 can suppress inflammation by negatively regulating classical and non-classical NF-κB pathways, thereby preventing tumorigenesis caused by chronic inflammation [15]. Previous researchers have found that the variant of NLRP12 can promote the occurrence of inflammatory diseases, such as systemic autoinflammatory diseases and episodic and refractory arthritis [13, 16, 17]. Furthermore, NLRP12 is a potential target for dengue virus, JEV, YFV, and ZIKV infection [18]. During inflammation, the MAPK pathway (including p38, JNK, ERK, and ERK5) and NF-кB pathway are activated [19, 20]. NLRP12 also plays a regulatory role in these pathways. Studies indicated that NLRP12(−/−) mice likely suffer from colon inflammation and colon cancer, and the progression of which activates the NF-кB and ERK pathways [19, 21]. Another study indicated that NLRP12 reduced the inflammation and proliferation of hepatocytes via the JNK pathway but not the p38, ERK, and NF-кB pathways [22, 23]. Moreover, 7-*O*-(2-(Propylamino)-2-oxoethyl) hesperetin promotes NLRP12 expression and then reduces inflammatory response through the NF-кB pathway [24]. However, no reports of NLRP12 in TNBC have been found at present.

This study aimed to explore the role of NLRP12 in TNBC. First, TNBC tissues and cells were collected to evaluate the expression of NLRP12, and results indicated that NLRP12 was lowly expressed in TNBC. Then, the NLRP12 expression was interfered to investigate the changes in cell function and the key protein expression in the NF-κB, ERK, p38, and JNK pathways. In addition, the results indicated that the inhibition of the NLRP12 activated the NF-κB pathway as well as increased cell proliferation, migration, and invasion. Therefore, PTD-p65-P1, an inhibitor of the NF-κB pathway, was used to explore whether the cell function can be reversed. Moreover, the findings in cells were observed in vivo.

## Materials and Methods

### Collection of TNBC Tissues and Immunohistochemistry (IHC)

A total of 30 pairs of TNBC tissues (cancerous group) and noncancerous tissues (noncancerous group) were collected from 2019 to 2021 in Shenzhen Longhua District Hospital. Our experiment was approved by the Committee of Shenzhen Longhua District Hospital, and informed consent was obtained from each patient. The study was conducted in accordance with the Declaration of Helsinki. TNBC tissues were sliced into 4 μm after being embedded in paraffin. Afterward, optimal tissues sections were selected for IHC staining. NLRP12 antibody was purchased from ABCAM (#ab105409).

### Cell Culture

MCF-10A, MCF-7, T47D, SKBR3, BT474, MDA-MB-436, HCC70, MDA-MB-231, and MDA-MB-157 were all purchased form CellCook (Guangzhou, China). They were kept in an 37°C incubator with 5% CO_2_ and amplified in different media: MCF-10A (DMEM + 5% horse serum + 10 μg/mL of insulin + 20 ng/mL of EGF + 0.5 μg/mL of hydrocortisone), MCF-7 (MEM + 10% FBS + 10 μg/mL of insulin + 1 × nonessential amino acid), T47D, BT474 (RPMI 1640 + 10% FBS + 10 μg/mL of insulin), SKBR3 (McCoy’s 5a + 10% FBS), MDA-MB-436 (L-15 + 10% FBS + 10 μg/mL of insulin + 16 μg/mL of glutathione), HCC70 (RPMI 1640 + 10%FBS), MDA-MB-231, and MDA-MB-157 (L-15 + 10% FBS).

### Cell Treatment

Control lentivirus (Ctl-lentivirus), NLRP12 lentivirus 1 (NLRP12 lentivirus #1), and 2 (NLRP12 lentivirus #2) were provided by General Biol (Anhui, China). In brief, the Sh-NLRP12-1 sequence (GTTAACAACTAAGAGAUAAUCAAAUCUGCCUTTTCTTCAAGAGAGCAGAUUUGAUUAUCUCUUCUTTTTTTCCTCGAG) and Sh-NLRP12-2 sequence (GTTAACAACTAGAAGUAUUCCUUCCUUUCUGTTTCTTCAAGAGAGAAAGGAAGGAAUACUUCUACTTTTTTCCTCGAG) were separately inserted into pLKO.1-U6-EF1a-copGFP-T2A-puro to produce lentivirus 1 and 2. The lentivirus was infected into MDA-MB-231 and MDA-MB-157 to interfere the NLRP12 expression in accordance with the instruction.

In investigating the role of NLRP12 in the NF-κB pathway, 150 μM PTD-p65-P1 (MCE, #HY-P1832) was added into the infected lentivirus cells for 16 h. Then, cells were collected for further analysis.

### Real-Time Quantitative PCR (qPCR)

TriQuick Reagent (Solarbio, #R1100) was used to isolate total RNA from tissues or cells. HiScript III RT SuperMix for qPCR (+gDNA wiper; Vazyme, Nanjing, China; #R323-01) was used to synthesize cDNA, and ChamQ Universal SYBR qPCR Master Mix (Vazyme; #Q711-02) was used for qPCR detection. The relative expression of NLRP12 was calculated using 2^−△△Ct^. GAPDH was the internal gene. The primer information was listed as follows: NLRP12-F: 5′-CCTGGGGAAGCATGGAGAAG-3′; NLRP12-R: 5′-ACCAGGTCCTCTCTCTGTCC-3′; GAPDH-F: 5′-GAGTCAACGGATTTGGTCGT-3′; GAPDH-R: 5′-GACAAGCTTCCCGTTCTCAG-3′.

### Western Blot

After the indicated treatment, cells were collected for Western blot. RIPA was used to isolate total protein. The BCA method was used to evaluate the concentration of total protein. Then, 20 μg of total protein was used for the detection of protein expression, following the standard protocol. The primary antibody information was listed as follows: NLRP12 (Abcam; #ab105409), GAPDH (Proteintech; #10494-1-AP), p-IκBb-α (ABclonal; #AP0707), IκBb-α (ABclonal; #A1187), p-ERK1/2 (ABclonal; #AP0974), ERK2 (ABclonal; #A0229), p-p38 MAPK (ABclonal; #AP0526), p38 MAPK (ABclonal; A14401), p-JNK1/2/3 (ABclonal; #AP0631), and JNK1/2/3 (ABclonal; A4867). HRP-conjugated Affinipure Goat Anti-Rabbit IgG(H + L) was used as a secondary antibody (Proteintech; #SA00001-2).

### Cell Function Detection

In our study, colony formation assay was used to evaluate proliferation in cells, whereas Transwell assay was used to detect migration and invasion. After the indicated treatment, cells were collected for colony formation and Transwell assay in accordance with the standard protocol.

### Xenograft Mouse Model

In our study, 10 specific-pathogen-free-grade nude mice (4 weeks old) were purchased from Cyagen Biosciences. They were divided into two groups (five mice per group): the MDA-MB-231-Ctl-lentivirus group and the MDA-MB-231-NLRP12-lentivirus group. MDA-MB-231 (3 × 10^6^ cells per mouse) were injected subcutaneously into nude mice for 2 weeks, and then 5 × 10^12^ copy lentivirus (100 μL) was injected into the tail vein for another 2 weeks. In the last 2 weeks, tumor volume was monitored two times a week. Afterward, mice were sacrificed by CO_2_, and tumors were collected for photo and weight measure. Tumors were used for further qPCR and Western blot analysis. Our animal experiment was approved by the Animal Ethics Committee of Shenzhen Longhua District Hospital.

### Statistical Analysis

SPSS and GraphPad 7.0 were used to analyze all data. Data was shown as mean ± standard deviation. Paired *T*-test was used to compare the IHC score difference. Student *T*-test was used to calculate the difference between two groups, and Analysis of Variance was used to calculate the difference among three or more groups. *P* < 0.05 indicates significant difference (*).

## Results

### NLRP12 was Lowly Expressed in TNBC Tissues and Cells

**Fig. 1:**
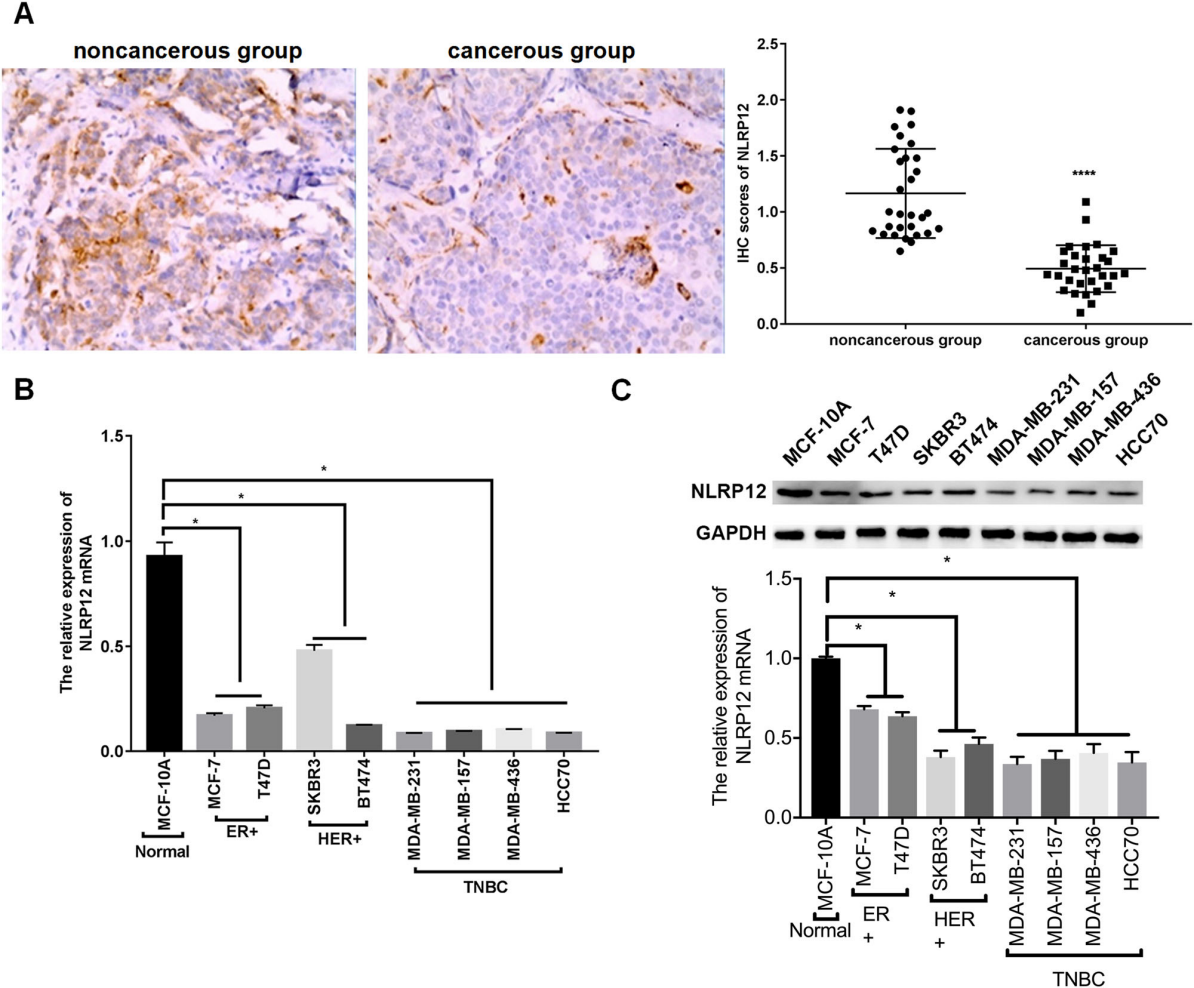
NLRP12 was lowly expressed in TNBC tissues and cells. **A** The expression of NLRP12 was determined using IHC assay in TNBC and paracancerous tissues. The upper panel shows representative pictures, and the lower panel shows quantitative analysis of IHC results in 30 pairs of tissues. **B** The expression of NLRP12 was analyzed by qPCR in normal breast cells (MCF-10A), ER+ breast cancer cells (MCF-7 and T47D), HER+ breast cancer cells (SKBR3 and BT474), and TNBC cells (MDA-MB-231, MDA-MB-157, MDA-MB-436, and HCC70). **C** The expression of NLRP12 was also examined in different breast cancer cells using Western blot, and NLRP12 expression was quantified

First, IHC was used to investigate the NLRP12 expression in 30 pairs of tissues, and the results indicated that NLRP12 was lowly expressed in the cancerous group compared with that in the noncancerous group (Fig. 1A). Then, normal, ER + , HER + , and TNBC cells were used to detect NLRP12 expression using qPCR, and the results implied that NLRP12 was restrained in the aforementioned cells compared with normal, ER + , and HER+ cells (Fig. 1B). In addition, the Western blot results indicated that the NLRP12 expression was declined in ER+ and HER+ cells relative to MCF-10A, particularly in MDA-MB-231 and MDA-MB-157 cells (Fig. 1C). Therefore, MDA-MB-231 and MDA-MB-157 cells were used for further study.

### Inhibition of NLRP12 Promoted the Proliferation, Migration, and Invasion in TNBC Cells

**Fig. 2:**
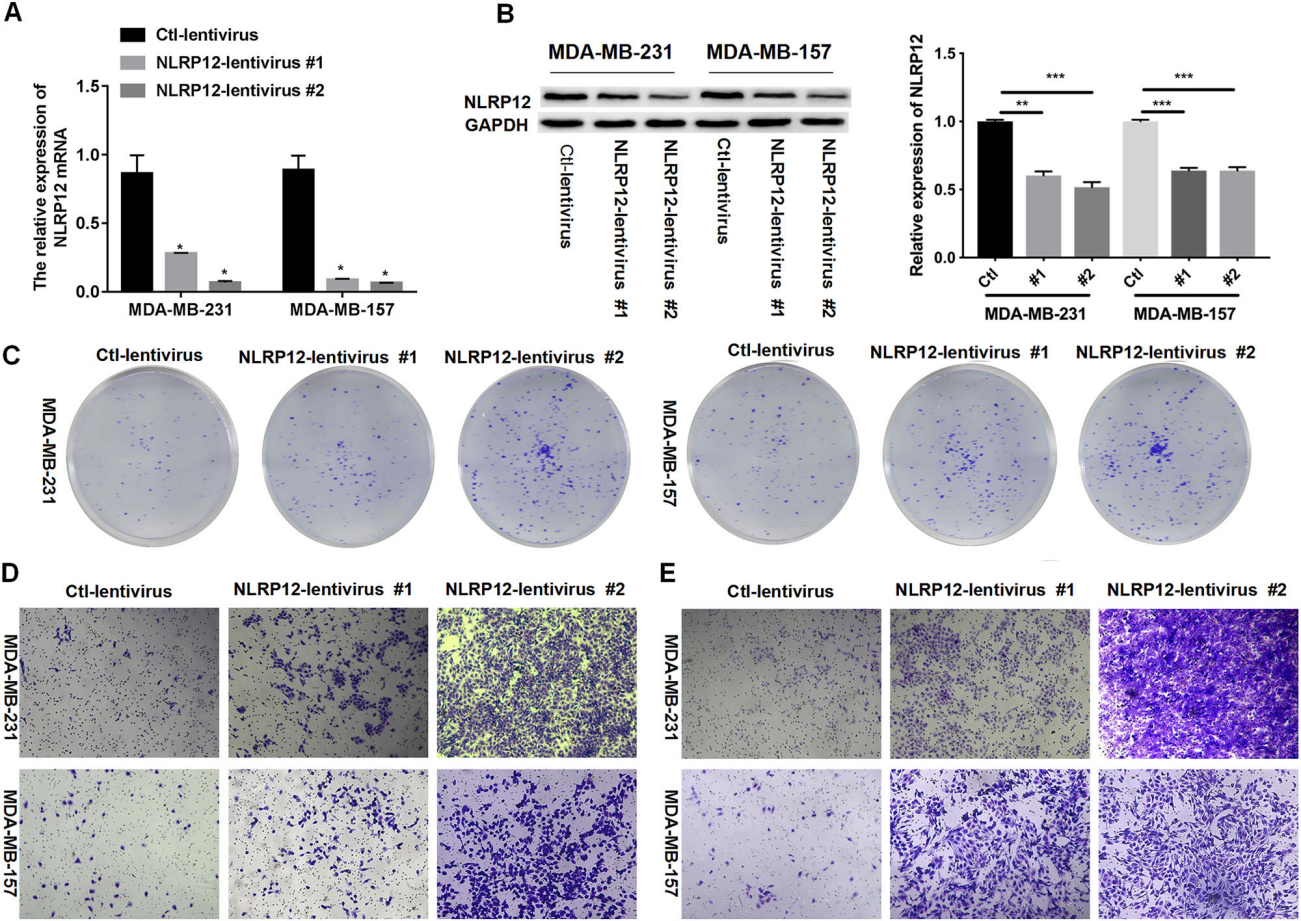
Inhibition of NLRP12 increased the proliferation, migration, and invasion in TNBC cells. MDA-MB-231 and MDA-MB-157 cells were transfected with NLRP12 lentivirus 1 and 2 for 48 h. **A** QPCR was performed to verify the change in NLRP12 expression in each group. **B** The change in NLRP12 expression was suggested via Western blot, and the relative expression of NLRP12 was counted. **C** Colony formation displayed the change in cell proliferation ability. Transwell assay analysis of cell migration (**D**) and cell invasion (**E**) in each group

In investigating the function of NLRP12 in TNBC cells, NLRP12 lentivirus 1 and 2 were infected with MDA-MB-231 and MDA-MB-157 cells for 48 h, respectively. The qPCR and Western blot results confirmed that NLRP12 lentivirus 1 and 2 effectively decreased the NLRP12 expression in MDA-MB-231 and MDA-MB-157 cells, and NLRP12 lentivirus 2 had better interference efficiency than NLRP12 lentivirus 1 (Fig. 2A, B). Compared with the Ctrl-lentivirus group, colony formation, migration, and invasion in cells were promoted by NLRP12 lentivirus 1 and NLRP12 lentivirus 2 (Fig. 2C–E). Collectively, decreased NLRP12 expression enhanced the proliferation, migration, and invasion of TNBC cells.

### Inhibition of NLRP12 Expression Enhanced the Proliferation, Migration, and Invasion in TNBC Cells by Activating the NF-κB Pathway

**Fig. 3:**
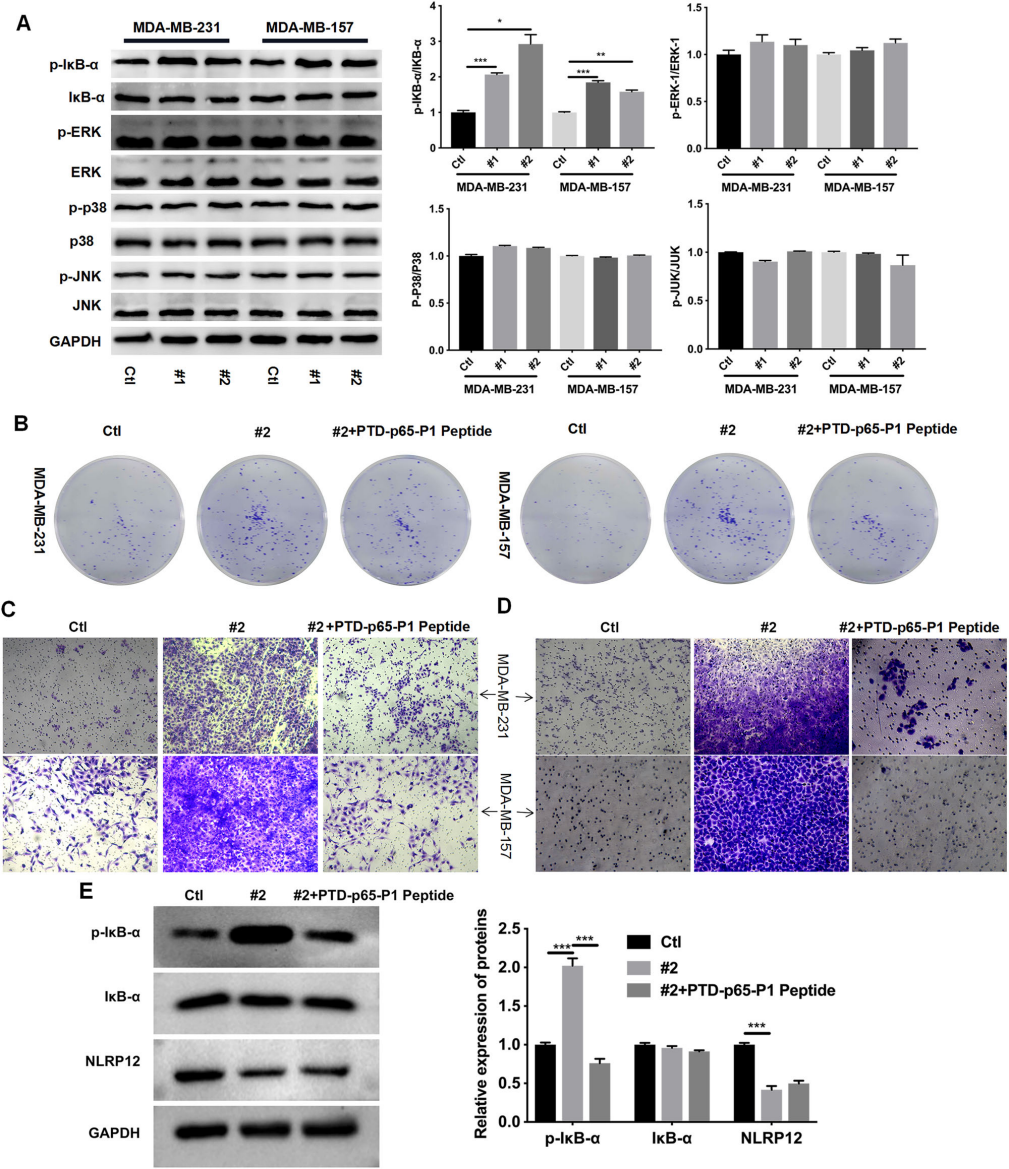
Inhibition of NLRP12 expression increased the proliferation, migration, and invasion in TNBC cells by activating the NF-κB pathway. **A** Western blot assay was applied to evaluate the changes in the expression of p-I-B-α, IκB-α, P-ERK, ERK, p-p38, p38, p-JNK, and JNK in MDA-MB-231 and MDA-MB-157 cells after transfection with NLRP12 lentivirus 1 and 2 for 48 h. In addition, the relative level of each protein was quantified on the basis of gray values. **B** Colony formation analysis of proliferation in MDA-MB-231 and MDA-MB-157 cells, which were treated with 150 μM PTD-p65-P1 peptide after transfection with NLRP12 lentivirus 2 for 48 h. After treatment with 150 μM PTD-p65-P1 peptide, cell migration (**C**) and invasion (**D**) were analyzed by applying Transwell assay in NLRP12 lentivirus 2-transfected MDA-MB-231 and MDA-MB-157 cells. **E** After processing with NLRP12 lentivirus 2 and PTD-p65-P1 peptide, the expression levels of p-I-B-α, IκB-α, and NLRP12 in MDA-MB-231 and MDA-MB-157 cells were also analyzed using Western blot

Previous studies indicated that NLRP12 regulates cancer development via the NF-κB, ERK, p38, and JNK pathways [19, 21, 22]. Therefore, the key proteins, including p-IκB-α, IκB-α, p-ERK, ERK, p-p38, p38, p-JNK, and JNK, were examined through Western blot. After interference of NLRP12 by lentivirus 1 and 2, only the p-IκBb-α expression was activated, and the other protein expression level remained unchanged (Fig. 3A). A previous study indicated that PTD-p65-P1 suppressed NF-κB activation [25]. Thus, whether the inhibition of NLRP12 expression could promote the function of TNBC cells was investigated by activating the NF-κB pathway. MDA-MB-231-NLRP12-lentivirus-2 and MDA-MB-157 cells-NLRP12-lentivirus-2 cells were treated with 150 μM PTD-p65-P1 for 16 h, and then colony formation and Transwell assay were conducted. The results indicated that PTD-p65-P1 repressed the proliferation, migration, and invasion in cells, which were enhanced by NLRP12 lentivirus 2 (Fig. 3B–D). In addition, Western blotting data indicated that p-I-B-α was notably upregulated in the LRP12-lentivirus-2 group relative to that in the control group, whereas the upregulation of p-I-B-α mediated by LRP12 silencing could also be dramatically reversed by PTD-p65-P1 in MDA-MB-231 and MDA-MB-157 cells. Moreover, PTD-p65-P1 did not change the downregulation of LRP12 mediated by LRP12 silencing in MDA-MB-231 and MDA-MB-157 cells (Fig. 3E). Collectively, the abovementioned results confirmed our hypothesis.

### Inhibition of NLRP12 Promoted Tumor Proliferation Speed and Activated the NF-κB Pathway

**Fig. 4:**
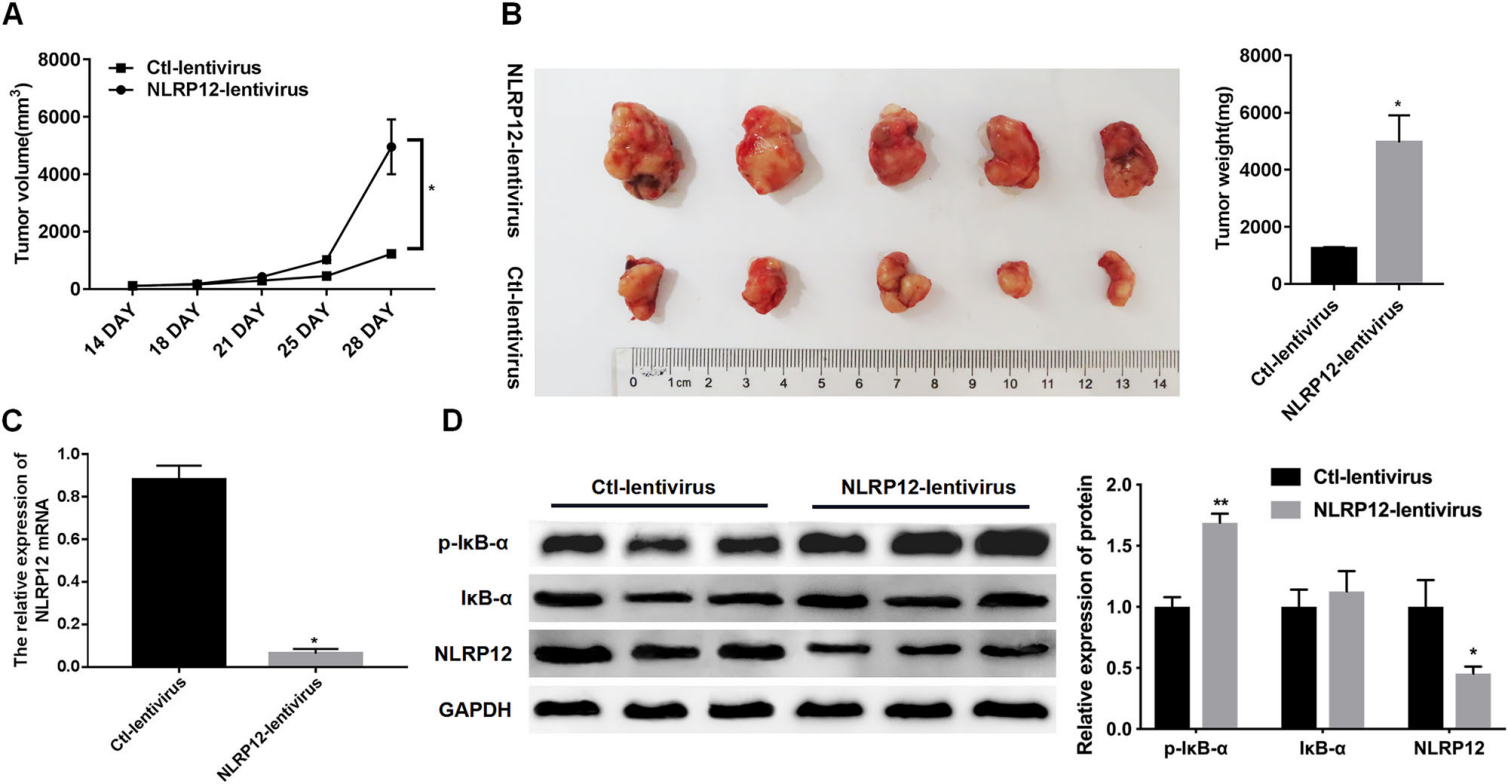
Inhibition of NLRP12 enhanced tumor proliferation speed and activated the NF-κB pathway. MDA-MB-231 (3 × 10^6^ cells) were injected subcutaneously into nude mice for 2 weeks, and then 5 × 10^12^ copy lentivirus (100 μL) was injected into the tail vein. **A** Tumor volume was monitored at 14, 18, 21, 25, and 28 days. **B** After 4 weeks, mice were sacrificed; tumor was collected, and the tumor weight was measured. **C** The expression of NLRP12 in tumor tissues was tested by qPCR. **D** The expression of p-IκBb-α, IκBb-α, and NLRP12 in tumor tissues was identified by Western blot, and quantitative analysis of each protein was presented

In addition, the role of NLRP12 in vivo was investigated. Tumor had evident difference between the MDA-MB-231-Ctl-lentivirus group and MDA-MB-231-NLRP12-lentivirus group in 28 days. The results showed that NLRP12-lentivirus promoted tumor volume and tumor weight compared with that in the Ctl-lentivirus group (Fig. 4A, B). Furthermore, NLRP12 expression was significantly declined in the NLRP12-lentivirus group relative to the Ctl-lentivirus group (Fig. 4C, D). Moreover, the expression level of p-IκBb-α increased after NLRP12 was inhibited (Fig. 4D).

## Discussion

Given the negative expression of ER, PR, and HER, TNBC lacks effective treatment. Therefore, its prognosis is poor, and metastasis rates and mortality are high [2, 26]. Finding a new therapy target may improve these phenomena. In our study, NLRP12 was lowly expressed in TNBC tissues and cells. The inhibition of NLRP12 increased the proliferation and metastasis in TNBC cells by activating the NF-κB pathway. Moreover, decreased NLRP12 expression promoted the tumor size and weight in vivo.

NLRP12 has a C-terminal leucine-rich repeat, an PYD, and a central NBD, and it plays a role in the formation of inflammasome in response to proliferation and inflammation. A previous study found that NLRP12 inflammasome induces inflammatory events during the development and progression of aggressive prostate cancer via regulating IL-1β and IL-18 [27]. NLRP12 deficiency promoted hepatocyte proliferation and increased inflammation in diethylnitrosamine induced hepatocellular carcinoma [22]. However, there is an interesting phenomenon between microglia and glioma. NLRP12 expression was high in glioma tissues, and the inhibition of NLRP12 decreased proliferation in glioma cells. For microglia, NLRP12 deficiency increased colony formation [28]. Therefore, NLRP12 may play a different role in different cancer types. In this study, NLRP12 was lowly expressed in TNBC, and the inhibition of NLRP12 expression enhanced proliferation, migration, and invasion abilities, which is consistent with the results in hepatocellular carcinoma.

In NLRP12-regulated inflammation, the MAPK pathway and NF-κB pathway are often activated. The MAPK pathway contains four cascades: p38 MAPK, ERK1/2, JNK, and ERK5 [29]. Among which, the p38 MAPK (p38), ERK1/2 (ERK), and JNK pathways are often observed in NLRP12-regulated inflammation of different diseases [11]. For example, overexpressed NLRP12 suppressed the phosphorylation of JNK, ERK1/2, and p38 in fibroblast-like synoviocytes of rheumatoid arthritis [30]. NLRP12 reduced the progression of hepatocellular carcinoma via suppressing JNK activation but not the NF-κB, ERK, and p38 pathways [22]. NLRP12 plays a protective role by negatively regulating the NF-κB pathway during periapical bone destruction to attenuate inflammation and osteoclastogenesis [31]. The NLRP12-mediated inhibition of the NF-kB pathway decreases hepatocyte apoptosis in alcohol-induced liver injury [32]. In addition, these two pathways are confirmed in innate immunity; host defense against *Brucella abortus* and *Pseudomonas aeruginosa*; osteoclast differentiation in bone metabolism, and obesity, [11, 33–36]. Collectively, NLRP12 serves as a negative regulator for the MAPK pathway and NF-κB pathway, but it may activate one or some of the pathways. Therefore, key proteins in the p38, ERK, JNK, and NF-κB pathways were determined after silencing of NLRP12. The results indicated that the inhibition of NLRP12 activated the NF-κB pathway but not the p38, ERK, and JNK pathways. Moreover, inhibiting the NF-κB pathway in PTD-p65-P1 reversed the effect of NLRP12 on cell proliferation, migration, and invasion. Furthermore, the xenograft mouse model confirmed the abovementioned findings.

However, the number of human tissues still low, and the upstream or downstream mechanism of NLRP12 remains unknown. Thus, more experiments should be conducted to find the upstream or downstream mechanism of NLRP12 from non-coding RNA and epigenetics, and more tissues should be collected to confirm the expression of NLRP12 and its potential mechanism.

## Conclusions

In this study, NLRP12 was downregulated in TNBC tissues and cells, and the results showed that the inhibition of NLRP12 could increase the proliferation, migration, and invasion in TNBC cells by activating the NF-κB pathway. These findings provided insights into TNBC therapy.
